# Phenylalanine modulates casein synthesis in bovine mammary epithelial cells by influencing amino acid transport and protein synthesis pathways

**DOI:** 10.3389/fnut.2025.1598191

**Published:** 2025-07-10

**Authors:** Yuanyuan Xing, Yixuan Luo, Mei Sun, Jing Yang, Shaoxiong Lin, Xiaojia Mu, Xiaoyu Niu, Dabiao Li, Yuanyuan Liu

**Affiliations:** ^1^College of Animal Science, Inner Mongolia Agricultural University, Hohhot, China; ^2^Key Laboratory of Animal Nutrition and Feed Science at Universities of Inner Mongolia Autonomous Region, Hohhot, China; ^3^National Center of Technology Innovation for Dairy, Hohhot, China; ^4^College of Science, Inner Mongolia Agricultural University, Hohhot, China

**Keywords:** phenylalanine, amino acid transport, amino acid metabolism, bovine mammary epithelial cells, casein synthesis, mTOR signaling pathway

## Abstract

The efficiency of phenylalanine (Phe) utilization for milk protein synthesis in dairy cows is limited, and its uptake and metabolic mechanisms in the mammary tissue remaining unclear. This study investigated the effects of Phe availability (0.07, 0.14, 0.28, and 0.56 mM) on amino acid metabolism and casein synthesis in bovine mammary epithelial cells (BMECs) cultured for 24 h. Results showed that α_S1_-casein, *β*-casein, and *κ*-casein expression peaked at 0.14 mM Phe (*p* < 0.05). At this optimal concentration, amino acid transporters (*SLC7A5*, *SLC7A8*, and *SLC38A2*) were upregulated, corresponding with enhanced uptake of Met, Ile, His, and Arg (*p* < 0.05). The mammalian target of rapamycin (mTOR) signaling pathway was activated as evidenced by increased phosphorylation of P70 S6 kinase (P70S6K) and mTOR (*p* < 0.05), while the general control nonderepressible 2 (GCN2) pathway was suppressed through reduced eukaryotic initiation factor 2α (eIF2α) phosphorylation (*p* < 0.05). As Phe concentration increased, its net uptake increased linearly (*P_linear_* < 0.05) while uptake efficiency decreased linearly (*P_linear_* < 0.05). High Phe concentration (0.56 mM) inhibited amino acid transporter expression and reduced uptake of Leu, Pro, and Tyr (*p* < 0.05). Additionally, Phe-to-Tyr conversion was dynamically regulated, with phenylalanine hydroxylase (PAH) activity inhibited at 0.07 mM Phe (*p* < 0.05) but enhanced at higher concentrations, concurrent with reduced exogenous Tyr uptake (*p <* 0.05). These findings show that casein synthesis in BMECs is optimal at 0.14 mM Phe, coinciding with enhanced expression of amino acid transporters and activation of protein synthesis pathways. In contrast, higher Phe concentrations (0.56 mM) are associated with reduced amino acid utilization efficiency. These observations suggest potential mechanisms by which Phe concentration may regulate milk protein synthesis in dairy cows.

## 1 Introduction

In recent years, the concept of precision protein nutrition has gained significant attention in dairy cattle feeding strategies, aiming to optimize milk protein yield while reducing nitrogen excretion and environmental impact (1). The protein requirements of dairy cows are essentially demands for amino acids, specifically essential amino acids (EAA (2);). The synthesis of milk protein depends critically on the availability and metabolic efficiency of EAA in mammary epithelial cells (3).

In corn-soybean meal-based diets, Lys and Met are traditionally considered the first and second limiting amino acids, respectively, while His often becomes the first limiting amino acid in silage-based diets. Consequently, extensive research has focused on these three amino acids and their effects on lactation performance (4, 5). With the emergence of the functional amino acid concept, increasing attention has also been directed toward Arg and branched-chain amino acids in dairy cow lactation (6, 7). However, despite continuing efforts to understand the role of various amino acids in milk protein synthesis, Phe, an essential amino acid for lactating dairy cows, has received comparatively limited research attention. This is particularly noteworthy because the mammary gland exhibits unique characteristics in handling Phe compared to other EAA. Studies have shown that the uptake efficiency of Phe by mammary glands is relatively low, with limited regulatory capacity. Doepel et al. (8) demonstrated that removing Phe from an amino acid mixture administered via abomasal infusion to cows fed low-protein diets significantly reduced mammary Phe uptake by 23.7% and decreased milk protein yield by 15.6%. This reduction was comparable to that observed in control groups receiving no amino acid supplementation. In contrast, the removal of Thr from the infusion did not affect its mammary uptake or milk protein production. A distinctive feature of mammary amino acid metabolism is the significant compensatory mechanism for most EAA. When the supply of limiting amino acids such as Lys and Met, or other EAA (Arg, Val, Leu, Ile) is reduced, the mammary gland can maintain their uptake and sustain milk protein synthesis by increasing uptake efficiency (8, 30). However, this compensatory capacity appears notably limited for Phe. Doelman et al. (9) found that in lactating dairy cows, abomasal infusion of EAA mixtures lacking specific amino acids reduced milk protein content. Notably, mixtures deficient in Phe caused a significant 23.6% decrease in milk protein yield compared to the complete EAA mixture. This reduction was more severe than those seen with Met-deficient mixtures (15.8% decrease) and Try-deficient mixtures (6.7% decrease). These findings suggest that the regulation of Phe uptake and metabolism may involve unique mechanisms that are still not fully understood. Additionally, due to the complex interactions among amino acids (31), the impact of varying Phe concentrations on the uptake and metabolism of other amino acids needs further elucidation.

Mammalian cells adapt to changes in intracellular amino acid levels and regulate protein synthesis via two pathways: the general control nonderepressible 2 (GCN2) and mechanistic target of rapamycin [mTOR; Pakos-Zebrucka et al. (10) and Saxton and Sabatini (11)]. Under conditions of amino acid deprivation, the accumulation of uncharged tRNA activates GCN2, which phosphorylates eIF2α, inhibiting protein synthesis. This also promotes activating transcription factor 4 (ATF4) translation, which induces genes for amino acid adaptation (12). In contrast, mTOR stimulates protein synthesis by inducing phosphorylation of ribosomal protein S6 kinase 1 (S6K1) and 4E-binding protein 1 under nutrient-rich conditions (13, 14). Studies have shown that in bovine mammary epithelial cells, the addition of various EAA either individually or in combination can stimulate the activation of the mTOR signaling pathway (1), while EAA deficiency leads to the inhibition of the mTOR signaling pathway and the activation of the GCN2 signaling pathway (32). However, the mechanisms by which Phe availability regulates casein synthesis at the cellular level are still unclear. It remains unknown whether Phe specifically activates these pathways and if there is an optimal concentration for maximizing protein synthesis efficiency.

To address these knowledge gaps, this study employed a bovine mammary epithelial cells (BMECs) model to examine the effects of Phe availability on amino acid uptake, metabolism, and casein synthesis. We hypothesized that Phe availability would regulate the uptake and metabolism of other amino acids, activate signaling pathways related to casein synthesis, and ultimately influence casein production in BMECs. The objective was to deepen our understanding of Phe utilization in bovine mammary cells and provide a scientific foundation for implementing precision amino acid nutrition strategies in dairy cattle.

## 2 Materials and methods

### 2.1 Ethics statement

This study was supported and approved by the Animal Ethics Committee of Inner Mongolia Agricultural University (No. NND2024007). All the animal experiments were performed in accordance with the National Standard Guidelines for Ethical Review of Animal Welfare (GB/T 35892–2018).

### 2.2 Culture of bovine mammary epithelial cells

The procedure for preparing primary BMECs was detailed in our previous study (15). In summary, BMECs were isolated from three multiparous lactating Holstein cows. The purified cells were cultured in plastic flasks (Corning, cat. no. CLS430639) using a growth medium consisting of Dulbecco’s Modified Eagle’s Medium/F12 Ham (DMEM/F12, Gibco, cat. no. A4192001) supplemented with 10% fetal bovine serum (ViVacell, cat. no. C04001-500), 100 U/mL penicillin–streptomycin (Gibco, cat. no. 15140122), 10 μg/mL hydrocortisone (Sigma–Aldrich, cat. no. H0135), 5 mg/mL insulin-transferrin-selenium (Gibco, cat. no. 41400045), and 10 ng/mL epidermal growth factor (Sigma–Aldrich, cat. no. E4127). The cultures were maintained at 37°C in a humidified atmosphere with 5% CO₂.

### 2.3 Experimental design

Subcultured BMECs were cultivated in the aforementioned growth medium without epidermal growth factor but with the addition of 100 ng/mL prolactin (Sigma-Aldrich, cat. no. L6520), at 37°C in a 5% CO₂ humidified atmosphere. When BMECs reached 85% confluence, they were randomly allocated into four treatment groups, each with six replicates. The inducing medium was formulated using a custom DMEM/F12 base medium (SunnCell, cat. no. SNM-004F-DZ) devoid of nine EAA and supplemented with varying concentrations of Phe (0.07 mM as the control, 0.14, 0.28, and 0.56 mM). The concentration of 0.07 mM approximates the Phe concentration in bovine arterial plasma supplying the mammary gland (16). The other EAA were added at the following concentrations: Lys (0.37 mM), Met (0.09 mM), His (0.33 mM), Arg (0.43 mM), Leu (0.61 mM), Ile (0.43 mM), Val (0.90 mM), and Thr (0.44 mM). These concentrations were derived from our previous research identifying optimal EAA levels for BMECs culture (16), which correspond to approximately 4 times the concentrations found in mammary arterial plasma of lactating dairy cows. After 24 h of incubation, cells were harvested to assess the effects of Phe concentrations on amino acid uptake, metabolism, and casein synthesis.

### 2.4 CCK-8 colorimetric assay

BMECs viability was assessed using the CCK-8 colorimetric assay (MedChemExpress, cat. no. HY-K0301). Subcultured BMECs were seeded into 96-well plates at a density of 1 × 10^5^ cells/well. The plates were incubated in a CO₂ incubator until the cells reached 85% confluence. At this point, inducing media containing different concentrations of Phe were added, and the cells were cultured at 37°C in a 5% CO₂ incubator for an additional 24 h. Four hours before the end of the culture period, 20 μL of CCK-8 solution was added to each well under light-protected conditions. At the conclusion of the experiment, the optical density (OD) at 490 nm was measured for each well using a fully automated microplate reader. The relative growth rate (RGR) of the cells was calculated as follows: RGR = (OD₄₉₀ nm of the experimental group)/(OD₄₉₀ nm of the control group).

### 2.5 Real-time quantitative PCR

The expression of amino acid transporters in BMECs was quantified using quantitative real-time PCR (qRT-PCR). Total RNA was extracted from BMECs using the Trizol reagent (Takara, cat. no. 9180) according to the manufacturer’s protocol. RNA quality and integrity were assessed using a NanoDrop spectrophotometer and 1% agarose gel electrophoresis. Total RNA was reverse-transcribed into cDNA using the PrimeScript RT reagent kit (Takara, cat. no. RR047A).qRT-PCR was performed with SYBR Premix Ex Taq II (Tli RNaseH Plus; Takara, cat. no. RR820A) in a 20 μL reaction volume. Primer sequences, product lengths, and annealing temperatures are listed in Table 1. The qRT-PCR conditions included an initial denaturation at 95°C for 30 s, followed by 40 cycles of 95°C for 5 s, annealing at 59 ~ 66°C for 30 s, and extension at 72°C for 30 s. The housekeeping genes *β*-actin (*ACTB*), glyceraldehyde-3-phosphate dehydrogenase (*GAPDH*), and ubiquitously expressed transcript (*UXT*) were used as internal controls. The geometric mean of these three housekeeping genes was calculated and used for normalization to improve the reliability of the qRT-PCR data. The relative changes in mRNA expression levels were calculated using the 2^-ΔΔCT^ method.

**Table 1:** Primer sequences used for quantitative real-time PCR.

Genes^1^	Primer sequences^2^	Accession no.	Length (bp)	Tm (°C)
*ACTB*	F:5’-GCCATGAAGCTGAAGATGAC-3’	NM_173979	195	62
	R:5’-CCTTCTGCAGCTCAGATATG-3’			
*GAPDH*	F:5’-GGGTCATCATCTCTGCACCT-3’	XM_001252479	110	60
	R:5’-GGTCATAAGTCCCTCCACGA-3’			
*UXT*	F:5’-CAGCTGGCCAAATACCTTCAA-3’	NM_005163	106	60
	R:5’-GTGTCTGGGACCACTGTGTCAA-3’			
*SLC7A5*	F:5’-GGTCAATGGGTCCCTCTTCAC-3’	NM_174613.2	112	60
	R:5’-GAGAGGATGGAGGGCAGATG-3’			
*SLC38A2*	F:5’-CACCCTGACAGTACCTGTTGTC-3’	NM_001082424.1	143	60
	R:5-CGATGCACACAGCAAATGAGTTAT-3’			
*SLC7A8*	F:5′- TGGAAGAAGCCGGACATCAAC-3’	NM_001192889.2	132	58
	R:5’-GCCCAGAACAGCAAGTAGATGAT-3’			
*SLC7A1*	F:5’-TCTATGCCTTCGTGGGCTTTGACT-3’	DQ399522.2	142	61
	R:5’-TCAGCCCAGAGAATTTGGAAGGCT-3’			
*SLC1A4*	F:5’-GGGTGACCTACAGCAAGTCC-3’	NM_001081577.1	115	60
	R:5’-CCTCCCGGAGAACGTAGGTA-3’			
*SLC3A2*	F:5’-CCCAACACCTCAGCATCTATAAACA-3’	EU118971.1	77	60
	R:5’-AGGGAGAGGAGGGAGTTACG-3’			
*ATF4*	F:5’-CAAGCTTGCATGCTAATTTGTCCC-3’	BC102236	87	60
	R:5’-TTGAGTCCTAGATCATGTCGAAGA-3’			

^1^Gene abbreviations: *ACTB* = β-actin; *GAPDH* = glyceraldehyde-3-phosphate dehydrogenase; *UXT* = ubiquitously expressed prefoldin like chaperone; *SLC7A5* = solute carrier family 7 member 5; *SLC38A2* = solute carrier family 38 member 2; *SLC7A8* = solute carrier family 7 member 8; *SLC7A1* = solute carrier family 7 member 1; *SLC1A4* = solute carrier family 1 member 4; *SLC3A2* = solute carrier family 3 member 2; *ATF4* = activating transcription factor 4.

^2^F, forward primer; R, reverse primer.

### 2.6 Western blotting

Western blotting was used to analyze the protein expression of casein and phosphorylation levels of key proteins involved in signaling pathways associated with casein synthesis. Total protein was extracted from BMECs using RIPA lysis buffer (Beyotime, cat. no. P0013B) containing protease and phosphatase inhibitors. Protein concentration was determined using the bicinchoninic acid (BCA) assay kit (Thermo Scientific, cat. no. 23225) according to the manufacturer’s instructions. Subsequently, 40 μg of protein from each sample was separated by SDS-PAGE and transferred onto polyvinylidene fluoride membranes (Millipore, Billerica, MA, cat. no. IPVH00010). The membranes were blocked at room temperature for 2 h using TBS blocking buffer (Beyotime, cat. no. P0228). Subsequently, the membranes were incubated overnight at 4°C with primary antibodies at appropriate dilutions (detailed in Table 2). The next day, the membranes were washed three times with TBST (5 min per wash) and incubated at room temperature for 2 h with fluorescence-labeled anti-rabbit IgG secondary antibodies (dilution 1:10,000; DyLight™ 800 4 × PEG Conjugate, Cell Signaling Technology, cat. no. S151S). The membranes were imaged using the Odyssey infrared imaging system (Odyssey Clx, LI-COR Biosciences, Lincoln, NE). The relative expression levels of target proteins were calculated as the ratio of the target protein’s signal intensity to that of *β*-actin.

**Table 2:** Information of antibodies used for western blotting.

Antibodies^1^	Suppliers	Dilution ratio	Molecular weight (kDa)
Rabbit polyclonal anti α_S1_-casein (ab166596)	Abcam	1:500	25
Rabbit polyclonal anti β-casein (orb10184)	Biorbyt	1:500	24
Rabbit polyclonal anti κ-casein (bs-10031R)	Bioss	1:500	19
Rabbit polyclonal anti mTOR (2972)	CST	1:1000	289
Rabbit polyclonal anti p-mTOR (Ser2448; 2,971)	CST	1:500	289
Rabbit monoclonal anti p70S6K (49D7)	CST	1:1000	70
Rabbit monoclonal anti p-p70S6K (Thr389; D5U1O)	CST	1:500	70
Rabbit monoclonal anti eIF2α (5324)	CST	1:500	38
Rabbit monoclonal anti p-eIF2α (Ser51; 3,398)	CST	1:500	38
Rabbit monoclonal anti β-Actin (4970)	CST	1:2000	42

^1^The antibodies targeted proteins involved in casein synthesis (α_S1_-casein, β-casein, κ-casein), the mTOR signaling pathway (mTOR, phosphorylated mTOR at Ser2448, p70S6K, phosphorylated p70S6K at Thr389), the GCN2 pathway (eIF2α, phosphorylated eIF2α at Ser51), and β-Actin, which served as a loading control.

### 2.7 Detection of amino acids content in BMECs culture media

The amino acids content in BMECs culture media was analyzed using an automated amino acid analyzer (Hitachi, Model L-8900). Before analysis, the culture supernatant was collected and treated with 8% sulfosalicylic acid (w/v) for overnight hydrolysis to remove proteins. The samples were centrifuged at 12,000 rpm for 10 min, and the supernatant was filtered through a 0.22 μm hydrophilic membrane before amino acid analysis. The following formulas were used to calculate the net amino acid uptake and net uptake efficiency of BMECs: (1) net amino acid uptake = amino acid content before incubation - amino acid content after incubation. (2) net amino acid uptake efficiency (%) = (net amino acid uptake / amino acid supply) × 100.

### 2.8 Detection of key enzyme activities related to EAA metabolism

The activities of key enzymes involved in the catabolism of EAA in BMECs were determined using ELISA kits (Jiangsu Enzyme Immunoassay Biotechnology) following the manufacturer’s protocols. The assessed enzymes included S-adenosylmethionine synthetase (SAMs, cat. no. MM-1044 V1), threonine dehydrogenase (TDH, cat. no. MM-1049 V1), phenylalanine hydroxylase (PAH, cat. no. MM-1053 V1), aminoadipate semialdehyde synthase (AASS, cat. no. MM-1057 V1), branched-chain amino acid transaminase (BCAT, cat. no. MM-51313O1), and arginase (ARG2, cat. no. MM-1045 V1).

### 2.9 Statistical analysis

Statistical analysis was conducted using SAS 9.2 software (SAS Institute Inc., Cary, NC, USA). Data were tested for normality with the Shapiro–Wilk test and for homogeneity of variance using Levene’s test, and all met the assumptions for parametric analysis. Results are presented as mean ± standard error of the mean (SEM) (*n* = 6). In the analysis of variance (ANOVA), replicate was treated as a random effect and treatment as a fixed effect. Differences between treatments were determined using Tukey’s multiple comparison test. Orthogonal polynomial contrasts were applied to evaluate linear and quadratic effects of treatment levels. Statistical significance was defined as *p* < 0.05.

## 3 Results

### 3.1 Effects of Phe availability on BMECs proliferation

**Figure 1:**
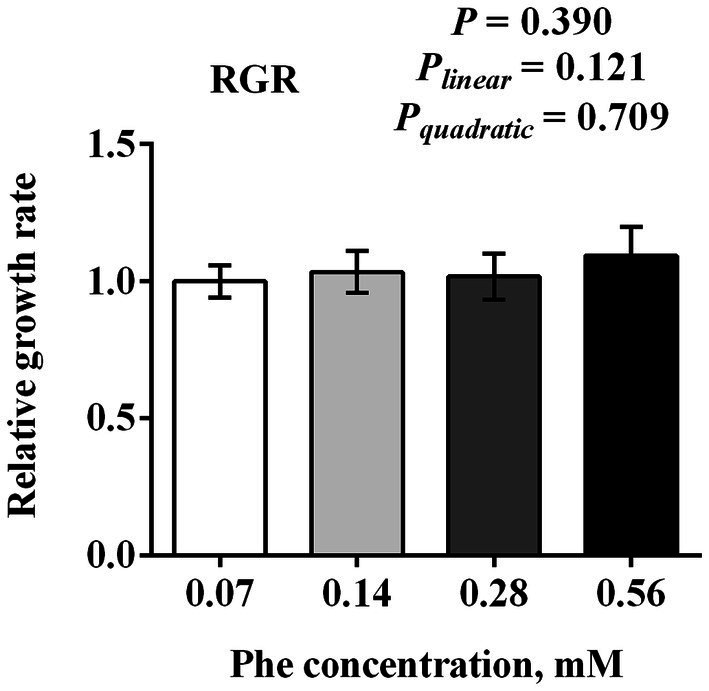
Effects of Phe availability on the proliferation of bovine mammary epithelial cells (BMECs). Purified BMECs were treated with varied concentration of Phe (0.07, 0.14, 0.28, and 0.56 mM) for 24 h. BMEC viability was assessed using the CCK-8 assay. Results are presented as means ± SEM (*n* = 6 replicates/treatment). Data were analyzed by one-way ANOVA followed by Tukey’s test for multiple comparisons. Values without a common letter differ (*p* < 0.05). Orthogonal polynomial contrasts were employed to assess linear and quadratic responses to increasing Phe levels.

As shown in Figure 1, increasing Phe concentrations from 0.07 to 0.56 mM did not significantly affect the proliferation of BMECs after 24 h of treatment (*p* = 0.390). No significant linear (*P_linear_* = 0.121) or quadratic (*P_quadratic_* = 0.709) responses were observed in relation to Phe concentration.

### 3.2 Effects of Phe availability on BMECs casein expression

**Figure 2:**
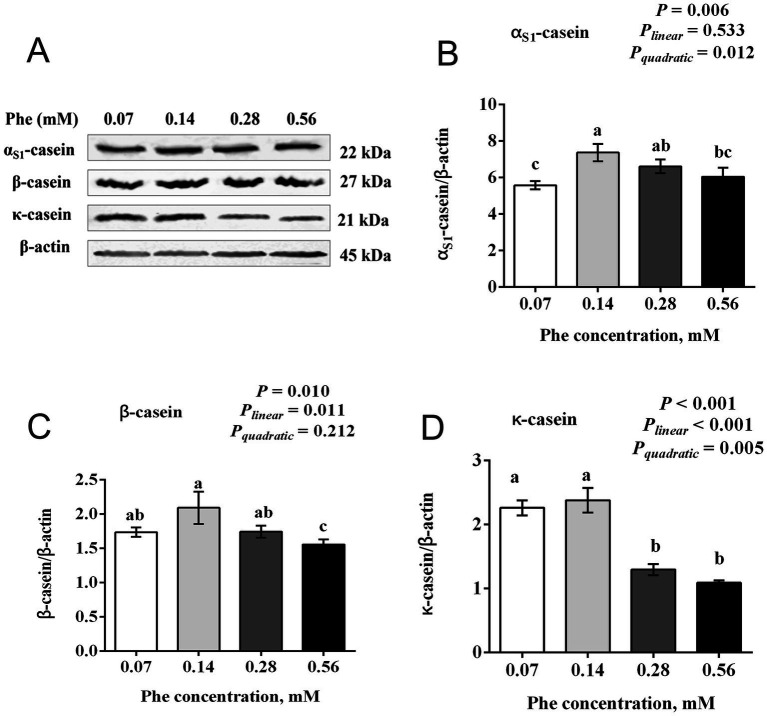
Effects of Phe availability on casein expression in bovine mammary epithelial cells (BMECs). Purified BMECs were treated with varied concentration of Phe (0.07, 0.14, 0.28, and 0.56 mM) for 24 h. **(A)** Western blotting analysis of α_S1_-casein, *β*-casein and κ-casein. Quantification of **(B)** α_S1_-casein, **(C)** β-casein and **(D)** κ-casein relative to β-actin. Results are presented as means ± SEM (*n* = 6 replicates/treatment). Data were analyzed by one-way ANOVA followed by Tukey’s test for multiple comparisons. Values without a common letter differ (*p* < 0.05). Orthogonal polynomial contrasts were employed to assess linear and quadratic responses to increasing Phe levels.

As shown in Figure 2, Phe concentration significantly influenced the expression of α_S1_-casein, *β*-casein, and *κ*-casein in BMECs. Expression levels of α_S1_-casein (*p* = 0.006), β-casein (*p* = 0.010), and κ-casein (*p* < 0.001) were highest at 0.14 mM Phe and lower at both 0.07 mM and higher concentrations (0.28 and 0.56 mM). α_S1_-casein exhibited a significant quadratic response (*P_quadratic_* = 0.012) to increasing Phe concentration. Similarly, β-casein showed a significant linear decrease (*Pₗᵢₙₑₐᵣ* = 0.011) as Phe concentration increased, with the lowest expression observed at 0.56 mM. κ-casein demonstrated both significant linear (*Pₗᵢₙₑₐᵣ* < 0.001) and quadratic (*P_quadratic_* = 0.005) responses, with expression markedly decreasing at concentrations above 0.14 mM.

### 3.3 Effects of Phe availability on amino acid net uptake by BMECs

**Figure 3:**
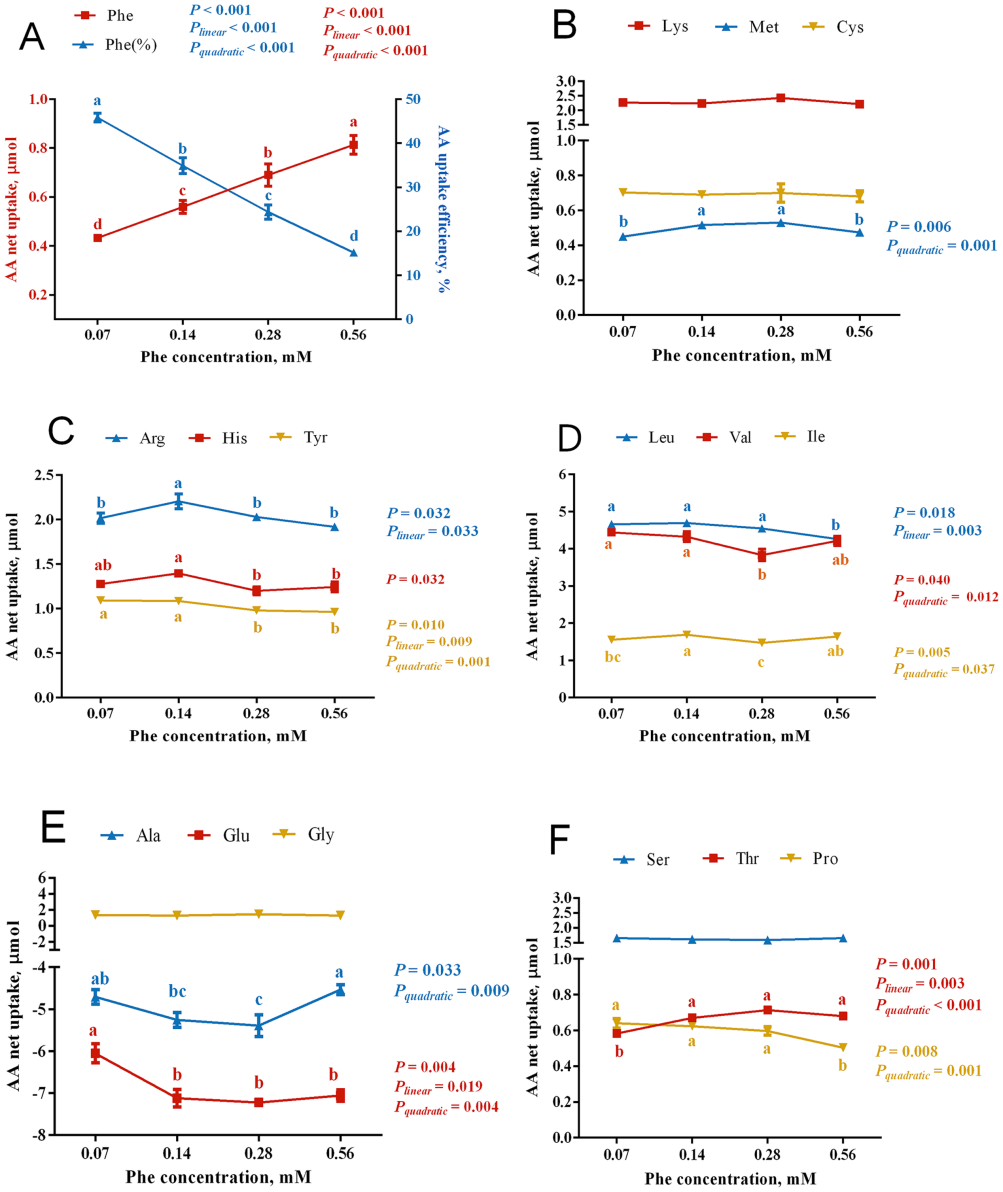
Effects of Phe availability on the net uptake of amino acids in bovine mammary epithelial cells (BMECs). Purified BMECs were treated with varied Phe concentrations (0.07, 0.14, 0.28, and 0.56 mM) for 24 h. Net AA uptake was calculated as AA content in medium before incubation minus after incubation. Phe uptake efficiency was calculated as Phe net uptake divided by Phe concentration. **(A)** Phe net uptake and uptake efficiency. **(B–F)** Net uptake of various amino acids: **(B)** Lys, Met, Cys; **(C)** Arg, His, Tyr; **(D)** Leu, Val, Ile; **(E)** Ala, Glu, Gly; **(F)** Ser, Thr, Pro. Results are presented as means ± SEM (*n* = 6 replicates/treatment). Data were analyzed by one-way ANOVA with Tukey’s test. Values without a common letter differ (*p* < 0.05). Orthogonal polynomial contrasts assessed linear and quadratic responses to increasing Phe levels.

As shown in Figure 3, increasing the Phe concentration in the culture medium led to a linear increase in the net uptake of Phe by BMECs (*P_linear_* < 0.001) and a linear decrease in Phe uptake efficiency (*P_linear_* < 0.001). Phe availability also significantly affected the net uptake of other amino acids.

Among EAA, the net uptake of Met exhibited a quadratic response to increasing Phe concentrations (*P_quadratic_* = 0.001), with significantly higher uptake in the 0.14 mM and 0.28 mM Phe groups than in the 0.07 mM and 0.56 mM Phe groups (*p* = 0.006). The net uptake of Thr showed both linear and quadratic increases with increasing Phe concentrations (*P_linear_* = 0.003, *P_quadratic_* < 0.001), with lower uptake in the 0.07 mM Phe group than in the other groups (*p* = 0.001). His uptake was significantly lower in the 0.28 mM and 0.56 mM Phe groups than in the 0.07 mM and 0.14 mM groups (*p* = 0.032). Arg uptake showed a significant linear decrease (*P_linear_* = 0.033) as Phe concentration increased, with greatest uptake in the 0.14 mM Phe group (*p* = 0.032).

The branched-chain amino acids showed varying responses to Phe concentration. Val uptake demonstrated a quadratic decrease (*P_quadratic_* = 0.012), with significantly lower uptake in the 0.28 mM Phe group compared to the 0.07 mM and 0.14 mM Phe groups (*p* = 0.040). Ile uptake exhibited a significant quadratic relationship (*P_quadratic_* = 0.037), with greater uptake in the 0.14 mM Phe group than in the 0.07 mM and 0.28 mM Phe groups (*p* = 0.005). Leu uptake decreased linearly (*P_linear_* = 0.003), with significantly lower uptake in the 0.56 mM Phe group compared to the other groups (*p* = 0.018).

Phe concentration also significantly affected the uptake of non-essential amino acids. The net efflux of Glu showed both linear and quadratic responses (*P_linear_* = 0.019, *P_quadratic_* = 0.004), with less efflux from the 0.07 mM Phe group than from the other groups (*p* = 0.004). Similarly, Ala exhibited a quadratic increase in net efflux (*P_quadratic_* = 0.009), with greater efflux in the 0.28 mM Phe group compared to the 0.07 mM and 0.56 mM Phe groups (*p* = 0.033). The net uptake of Tyr decreased both linearly and quadratically (*P_linear_* = 0.009, *P_quadratic_* = 0.001) with increasing Phe, with significantly greater uptake in the 0.07 mM and 0.14 mM Phe groups than in the 0.28 mM and 0.56 mM Phe groups (*p* = 0.010). Pro uptake decreased quadratically (*P_quadratic_* = 0.001), with significantly lower uptake in the 0.56 mM Phe group compared to the other groups (*p* = 0.008).

### 3.4 Effects of Phe availability on mRNA expression of amino acid transporters in BMECs

**Figure 4:**
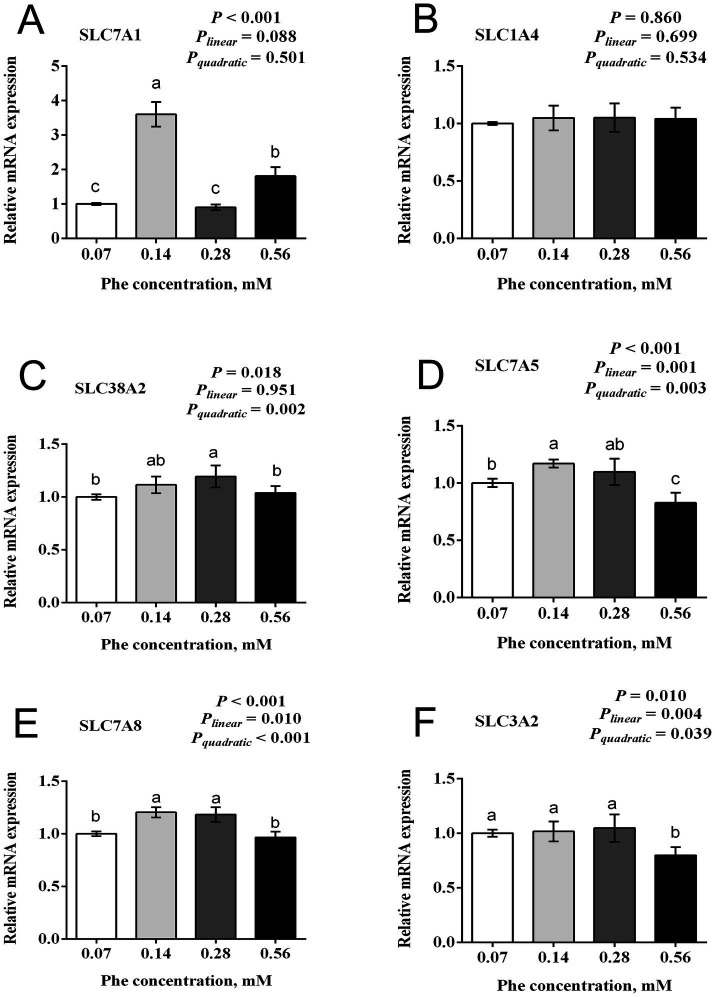
Effects of Phe availability on the mRNA expression of amino acid transporters in bovine mammary epithelial cells (BMECs). Purified BMECs were treated with varied Phe concentrations (0.07, 0.14, 0.28, and 0.56 mM) for 24 h. Relative mRNA expression of amino acid transporters: **(A)** SLC7A1 (Solute Carrier Family 7 Member 1, cationic amino acid transporter-1), **(B)** SLC1A4 (Solute Carrier Family 1 Member 4, neutral amino acid transporter), **(C)** SLC38A2 (Solute Carrier Family 38 Member 2, sodium-coupled neutral amino acid transporter 2), **(D)** SLC7A5 (Solute Carrier Family 7 Member 5, large neutral amino acid transporter small subunit 1), **(E)** SLC7A8 (Solute Carrier Family 7 Member 8, large neutral amino acid transporter small subunit 2), and **(F)** SLC3A2 (Solute Carrier Family 3 Member 2, 4F2 cell-surface antigen heavy chain). Results are presented as means ± SEM (*n* = 6 replicates/treatment). Data were analyzed by one-way ANOVA with Tukey’s test. Values without a common letter differ (*p* < 0.05). Orthogonal polynomial contrasts assessed linear and quadratic responses to increasing Phe levels.

Figure 4 illustrates the effects of Phe availability on the mRNA expression of amino acid transporters in BMECs. Several transporters showed the greatest expression at moderate Phe concentrations followed by decreased expression at higher concentrations. The expression of *SLC7A1* showed significant differences across treatments (*p* < 0.001), with peak expression at 0.14 mM Phe, significantly higher than all other groups, while the 0.56 mM group showed intermediate expression. Similarly, the expression of *SLC7A5* exhibited both linear and quadratic responses (*P_linear_* = 0.001, *P_quadratic_* = 0.003, *p* < 0.001), with the greatest expression at 0.14 mM Phe. The expression of *SLC38A2* showed a significant quadratic response (*P_quadratic_* = 0.002, *p* = 0.018), with peak expression at 0.28 mM Phe, significantly higher than at 0.07 mM and 0.56 mM Phe concentrations. The expression of *SLC7A8* and *SLC3A2* displayed similar patterns with significant linear and quadratic responses. The expression of *SLC7A8* showed significantly higher expression at 0.14 mM and 0.28 mM Phe compared to 0.07 mM and 0.56 mM Phe (*P_linear_* = 0.010, *P_quadratic_* < 0.001, *p* < 0.001). The expression of *SLC3A2* maintained high expression at 0.07 mM, 0.14 mM, and 0.28 mM Phe, with a significant decrease at 0.56 mM Phe (*P_linear_* = 0.004, *P_quadratic_* = 0.039, *p* = 0.010). Overall, five amino acid transporter genes examined showed significant responses to Phe concentration, typically with greatest expression at moderate Phe levels (0.14 mM or 0.28 mM) and decreased expression at the greatest Phe concentration (0.56 mM).

### 3.5 Effects of Phe availability on the mTOR signaling pathway in BMECs

**Figure 5:**
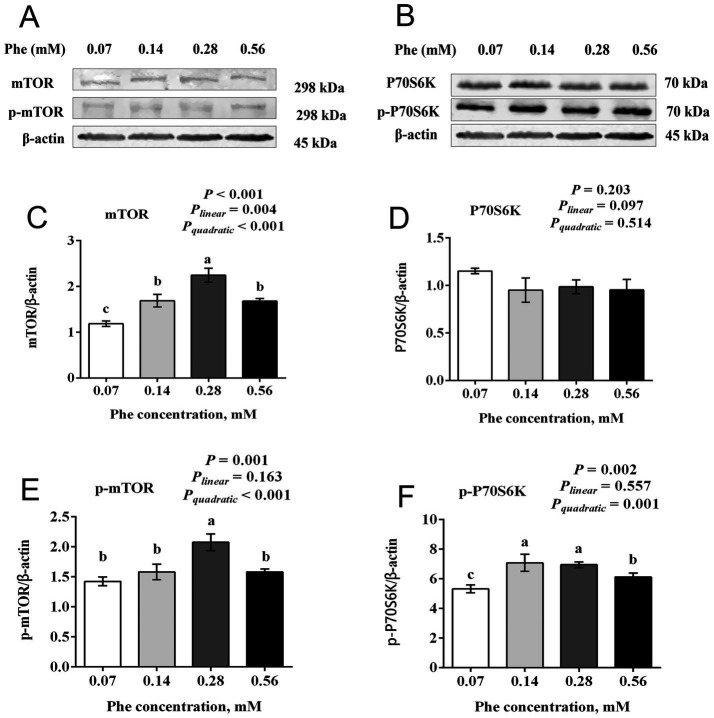
Effects of Phe availability on the expression of key proteins in the mTOR signaling pathway in bovine mammary epithelial cells (BMECs). Purified BMECs were treated with varied Phe concentrations (0.07, 0.14, 0.28, and 0.56 mM) for 24 h. **(A,B)** Western blotting analysis of mTOR, phosphorylated mTOR (p-mTOR), P70S6K, and phosphorylated P70S6K (p-P70S6K). Quantification of **(C)** mTOR, **(D)** P70S6K, **(E)** p-mTOR, and **(F)** p-P70S6K relative to β-actin. Results are presented as means ± SEM (*n* = 6 replicates/treatment). Data were analyzed by one-way ANOVA with Tukey’s test. Values without a common letter differ (*p* < 0.05). Orthogonal polynomial contrasts assessed linear and quadratic responses to increasing Phe levels.

As shown in Figure 5, increasing Phe concentrations in the culture medium affected the expression of the mTOR signaling pathway. Both total protein expression and phosphorylation levels of mTOR at Ser2448 showed a quadratic increase with increasing Phe concentrations (*P_quadratic_* < 0.001). The total protein expression and phosphorylation levels of mTOR at Ser2448 in the 0.28 mM Phe group were higher than in the other groups (*p* < 0.001 and *p* = 0.001, respectively). The total protein expression of mTOR in the 0.07 mM Phe group was lower than in the other groups (*p* < 0.001). Phosphorylation levels of P70 S6 kinase (P70S6K) at Thr389 showed a quadratic increase (*P_quadratic_* = 0.001). The phosphorylation levels of P70S6K at Thr389 in the 0.14 mM and 0.28 mM Phe groups were higher than in the 0.07 mM and 0.56 mM Phe groups (*p* = 0.002).

### 3.6 Effects of Phe availability on the expression of the GCN2 signaling pathway in BMECs

**Figure 6:**
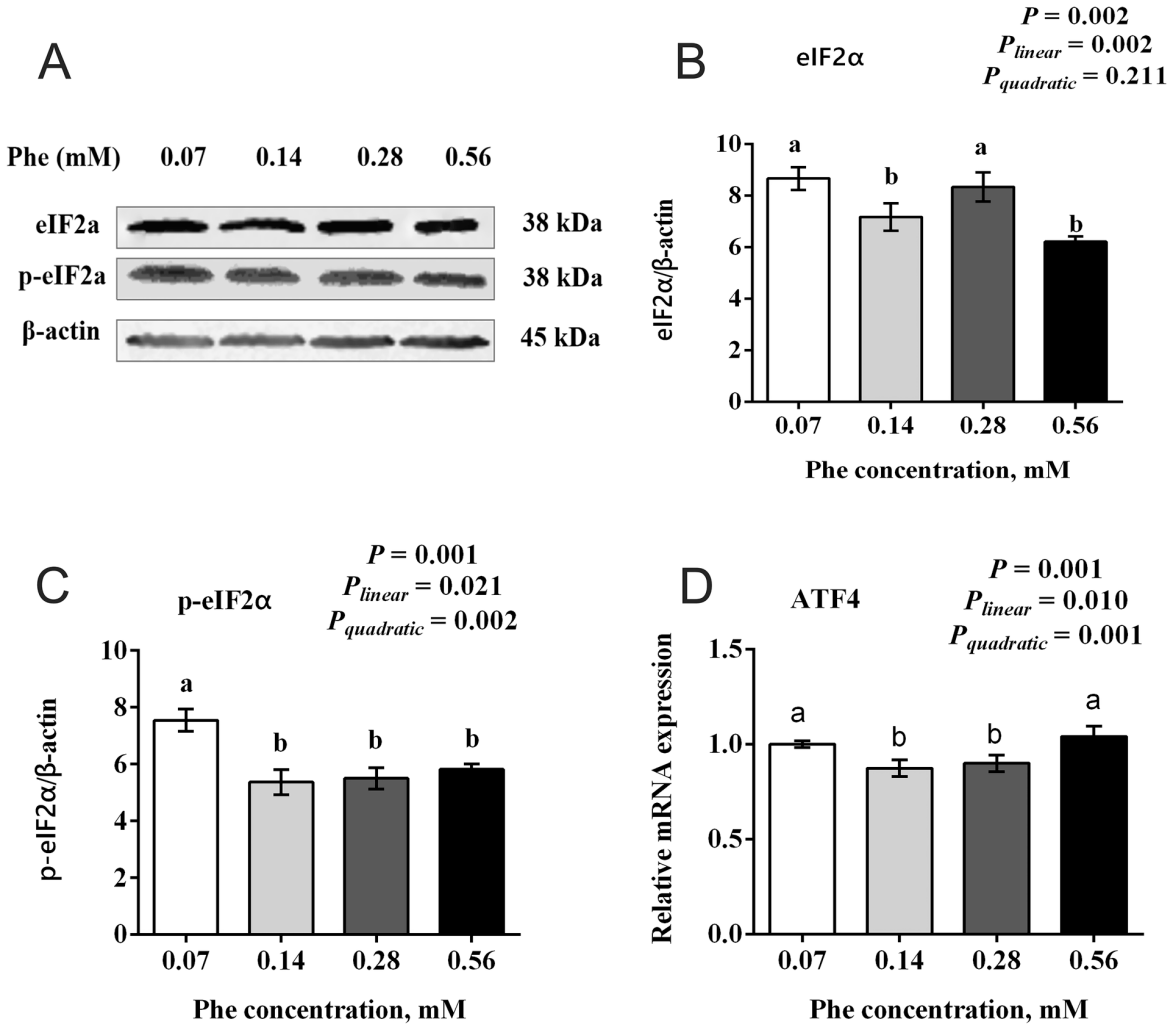
Effects of Phe availability on the expression of key proteins and genes in the GCN2 signaling pathway in bovine mammary epithelial cells (BMECs). Purified BMECs were treated with varied Phe concentrations (0.07, 0.14, 0.28, and 0.56 mM) for 24 h. **(A)** Western blotting analysis of eIF2α and phosphorylated eIF2α (p-eIF2α). Quantification of **(B)** eIF2α and **(C)** p-eIF2α relative to β-actin. **(D)** Relative mRNA expression of ATF4. Results are presented as means ± SEM (*n* = 6 replicates/treatment). Data were analyzed by one-way ANOVA with Tukey’s test. Values without a common letter differ (*p* < 0.05). Orthogonal polynomial contrasts assessed linear and quadratic responses to increasing Phe levels.

As shown in Figure 6, increasing Phe concentrations in the culture medium affected the expression of key proteins and genes in the GCN2 signaling pathway. The total protein expression of eIF2α showed a significant linear decrease with increasing Phe concentrations (*P_linear_* = 0.002, *p* = 0.002). The expression of eIF2α in the 0.07 mM and 0.28 mM Phe groups was higher than in the 0.14 mM and 0.56 mM Phe groups (*p* = 0.002). The phosphorylation level of eIF2α and the mRNA expression of *ATF4* both showed quadratic responses to increasing Phe concentrations (*P_quadratic_* = 0.002 and 0.001, respectively). The phosphorylation level of eIF2α was higher in the 0.07 mM Phe group than in all other groups (*p* = 0.001). Similarly, the mRNA expression of *ATF4* was higher in the 0.07 mM and 0.56 mM Phe groups compared to the 0.14 mM and 0.28 mM Phe groups (*p* = 0.001).

### 3.7 Effects of Phe availability on the activity of key enzymes involved in EAA catabolism in BMECs

**Figure 7:**
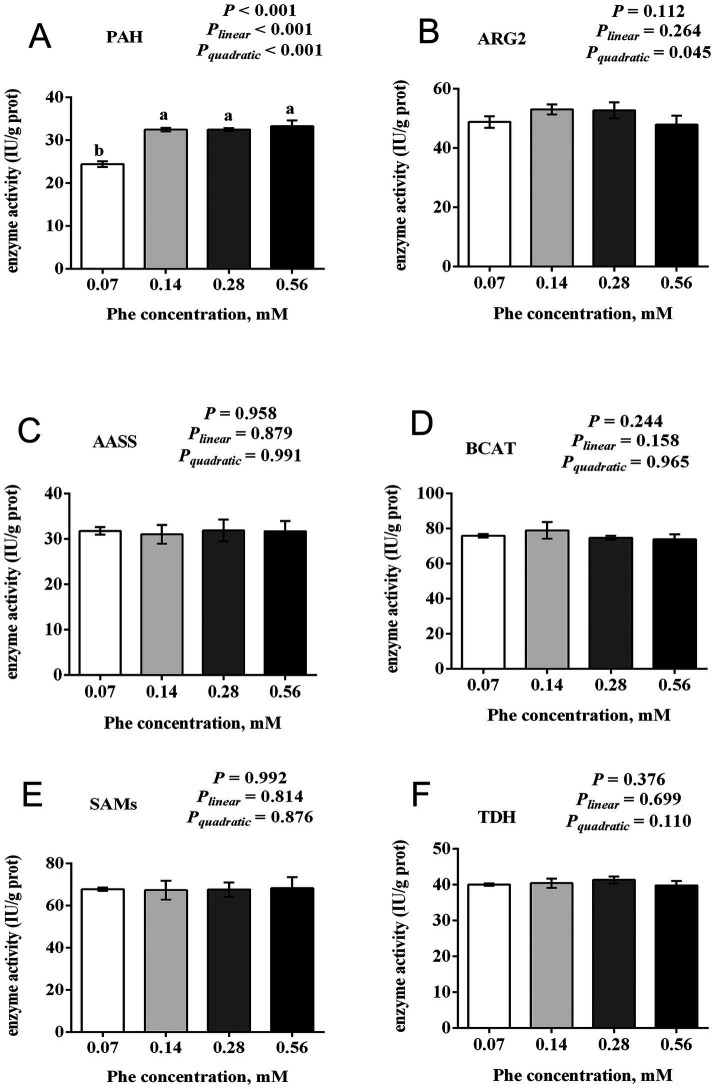
Effects of Phe availability on the activity of key enzymes in EAA catabolism in bovine mammary epithelial cells (BMECs). Purified BMECs were treated with varied Phe concentrations (0.07, 0.14, 0.28, and 0.56 mM) for 24 h. The enzymatic activities of **(A)** phenylalanine hydroxylase (PAH), **(B)** arginase 2 (ARG2), **(C)** argininosuccinate synthase (AASS), **(D)** branched-chain amino acid transaminase (BCAT), **(E)** S-adenosylmethionine synthetase (SAMs), and **(F)** threonine dehydrogenase (TDH) were measured. Results are presented as means ± SEM (*n* = 6 replicates/treatment). Data were analyzed by one-way ANOVA with Tukey’s test. Values without a common letter differ (*p* < 0.05). Orthogonal polynomial contrasts assessed linear and quadratic responses to increasing Phe levels.

As shown in Figure 7, increasing Phe concentrations in the culture medium affected the activities of key enzymes involved in EAA catabolism. The activity of PAH was reduced in the 0.07 mM Phe group (*p* < 0.001), while no differences in PAH activity were observed among the other groups. The activity of ARG2 increased quadratically with increasing Phe availability (*P_quadratic_* = 0.045). In contrast, the activities of AASS, BCAT, SAMs, and TDH showed no differences among the Phe treatment groups (*p* > 0.05).

## 4 Discussion

### 4.1 Effects of Phe availability on casein synthesis and cells proliferation in BMECs

As an essential amino acid for dairy cows, Phe must be supplied through the diet to sustain milk protein synthesis during lactation. Doepel et al. (8) demonstrated the importance of Phe by supplementing low-protein diet-fed cows with a mixed amino acid solution via abomasal infusion. Removing Phe from the solution reduced mammary uptake of Phe by 23.7% and decreased milk protein yield by 15.6%, highlighting the critical role of Phe availability in milk protein synthesis. In our study, increasing Phe concentration from 0.07 mM to 0.56 mM resulted in a quadratic increase in the expression of α_S1_-casein and *κ*-casein in BMECs, with the highest expression observed at 0.14 mM Phe. Similarly, the expression of *β*-casein also peaked at this concentration, suggesting that 0.14 mM is the optimal Phe level for casein synthesis in BMECs under the tested conditions. Furthermore, Guo et al. (17) reported that Phe activated the mTOR signaling pathway to upregulate 4E-binding protein 1 expression and reduces the phosphorylation level of eIF2α, thereby promoting protein synthesis. Our results align with these findings, showing that the total protein expression of mTOR, its phosphorylation levels, and the phosphorylation of P70S6K are consistent with the trends in casein expression. Conversely, eIF2α phosphorylation and *ATF4* mRNA expression exhibits opposite trends. These results suggest that optimal Phe concentrations may promote casein synthesis in BMECs partly by activating the mTOR and inhibiting the GCN2 signaling pathway. This highlights the potential importance of maintaining proper Phe levels to enhance milk protein production while avoiding disruptions to cellular regulatory processes.

Interestingly, our study found that increasing Phe concentrations from 0.07 to 0.56 mM did not significantly affect BMECs proliferation. This suggests that while Phe availability critically influences protein synthesis pathways and casein production, it may not directly regulate cell proliferation within this concentration range. This finding aligns with the specialized function of mammary epithelial cells during lactation, where resources are primarily directed toward milk protein synthesis rather than cell proliferation (18).

### 4.2 Effects of Phe availability on amino acid uptake and metabolism in BMECs

#### 4.2.1 Effects of Phe availability on its uptake and metabolism in BMECs

Amino acid uptake by the mammary gland is a complex physiological process. Studies have shown that approximately 90% of the amino acids taken up by mammary gland are ultimately used for milk protein synthesis, indicating that the net amino acid uptake by the mammary gland is primarily driven by the demands of milk protein synthesis. Mammary tissue can flexibly adjust amino acid uptake and utilization patterns based on the physiological state of the animal and the levels of amino acid supply (19). Lapierre et al. (20) reported that when His supply was insufficient, the mammary gland compensates by increasing His utilization efficiency to maximize milk protein synthesis. In our study, as Phe availability increased from 0.07 mM to 0.56 mM, its uptake increased linearly, while uptake efficiency decreased linearly, suggesting that BMECs can dynamically regulate Phe uptake efficiency. Interestingly, while Phe uptake increased linearly, casein synthesis peaked at 0.14 mM Phe. This indicated that additional Phe uptake beyond this concentration was not translated into greater casein production.

Regarding Phe metabolism in the mammary gland, previous studies have shown that Phe can be converted into Tyr, though the reported conversion rates vary (21, 22). Our results revealed that increased Phe uptake enhances the activity of PAH, the key enzyme catalyzing this conversion. Concurrently, Tyr uptake by BMECs significantly decreased in the 0.28 mM and 0.56 mM Phe groups. Based on these findings, we propose that the conversion of Phe to Tyr in BMECs is dynamically regulated: as intracellular Phe levels increase, its conversion to Tyr is upregulated, thereby reducing the requirement for exogenous Tyr. This dynamic regulatory mechanism not only explains the variability in reported Phe-to-Tyr conversion rates but is also supported by previous studies. For example, Jorgensen and Larson (23) demonstrated that bovine mammary tissue cultured *in vitro* could synthesize casein even in the absence of Tyr. This provides further evidence that mammary epithelial cells can modulate Phe conversion to Tyr to adapt to varying nutritional conditions.

#### 4.2.2 Interactive effects of Phe with other amino acids in BMECs

Amino acids in mammary epithelial cells are transported by membrane transporters. These transporters are essential for amino acid uptake and utilization in the mammary gland, particularly during lactation, when the demand for amino acids to support milk protein synthesis is high. Changes in the supply level of one amino acid can significantly affect the uptake and metabolism of others through shared transport systems (24). For example, cationic amino acid transporter 1 (encoded by *SLC7A1*) transports cationic amino acids such as Lys, Arg, and His. Sodium-coupled neutral amino acid transporter 2 (encoded by *SLC38A2*) and Ala, Ser, and Cys transporter 1 (encoded by *SLC1A4*) transport neutral amino acids like Ala, Gly, Ser, and Cys. The large neutral amino acid transporter1/4F2 cell-surface antigen heavy chain complex (encoded by *SLC7A5* and *SLC3A2*, respectively) facilitates the uptake of large neutral amino acids, including BCAA (Leu, Ile, Val), Phe, and Met. Similarly, the large neutral amino acid transporter 2/4F2 cell-surface antigen heavy chain complex complex (encoded by *SLC7A8* and *SLC3A2*, respectively) transports glutathione and most neutral amino acids (25).

In our study, with Phe levels increased from the optimal concentration for casein synthesis (0.14 mM) to a high level (0.56 mM), the uptake of Met, Leu, His, and Pro decreased, likely due to competition within shared neutral amino acid transport systems. The net uptake of the cationic amino acid Arg also declined at 0.56 mM Phe, accompanied by suppressed mRNA expression of amino acid transporters *SLC7A1*, *SLC7A5*, *SLC7A8*, and *SLC3A2*. These findings suggest that excessive Phe may inhibit the uptake of neutral amino acids through competitive inhibition and broadly suppress amino acid uptake by downregulating transporter expression. In contrast, at the optimal Phe concentration for casein synthesis (0.14 mM), the net uptake of Met, Ile, His, and Arg significantly increased. Similar results have been reported previously: infusing a combination of Met, Lys, and His into lactating cows improved the net uptake of Lys and Ile, enhanced nitrogen utilization efficiency, and increased milk protein yield by 84 g/day (26, 27). These results highlight the importance of a balanced amino acid supply to enhance uptake efficiency and promote milk protein synthesis. When the supply of an essential amino acid is insufficient, other amino acids cannot be effectively utilized for protein synthesis. For example, Tian et al. (28) found that a Leu deficiency increased plasma levels of Ile and Val but significantly reduced milk protein yield. Similarly, Lapierre et al. (20) reported that insufficient His supply increased oxidative loss of Leu in mammary tissue. In our study, a low Phe concentration (0.07 mM) led to reduced uptake of Thr and Met and significant suppression of α_S1_-casein expression, indicating that 0.07 mM Phe is inadequate for efficient casein synthesis.

Interestingly, we observed that the optimal Phe concentration range for the synthesis of the three types of casein is not identical. For example, compared to the 0.14 mM Phe group, the 0.07 mM Phe group significantly reduced the expression of α_S1_-casein, while the expression levels of *β*-casein and *κ*-casein showed no significant differences between these two groups. Additionally, compared to the 0.14 mM Phe group, the 0.28 mM Phe group exhibited significantly reduced uptake of Ile, Val, and His, as well as decreased expression of κ-casein, but the expression levels of α_S1_-casein and β-casein were not significantly affected. We speculate that this phenomenon may result from differences in amino acid composition among casein types (29). As a result, variations in the amino acid uptake patterns by mammary epithelial cells may influence the composition of casein.

## 5 Conclusion

This study demonstrates that Phe concentration significantly affects casein synthesis in BMECs. Casein synthesis peaks at 0.14 mM Phe, coinciding with optimal activation of the mTOR pathway and suppression of the GCN2 pathway. This concentration also promotes amino acid transporter expression, enhancing the uptake of key amino acids including Met, Ile, His, and Arg. In contrast, high Phe concentrations (0.56 mM) are associated with decreased amino acid transporter expression, lower uptake of multiple amino acids, and decreased casein synthesis. These findings improve our understanding of Phe’s role in milk protein synthesis and provide a molecular basis for optimizing Phe supply in precision amino acid nutrition strategies for dairy cows.

## Data Availability

The original contributions presented in the study are included in the article/supplementary material, further inquiries can be directed to the corresponding author/s.
